# Nonrelevant quantum levels effecting on the current in 2-well terahertz quantum cascade lasers

**DOI:** 10.1038/s41598-022-22396-6

**Published:** 2022-10-17

**Authors:** Li Wang, Tsung-Tse Lin, Ke Wang, Hideki Hirayama

**Affiliations:** 1 THz Quantum Device Team, RIKEN Center for Advanced Photonics, 519-1399 Aramaki-aza Aoba, Aoba-ku, Sendai, 980-0845 Japan; 2 School of Electronics Science and Engineering, Nanjing University, 163 Xianlin Street, Qixia District, Nanjing, 210046 China

**Keywords:** Optics and photonics, Lasers, LEDs and light sources, Semiconductor lasers

## Abstract

Recent renewed operating temperatures in terahertz quantum cascade lasers emphasize on narrowing the periodic length in a 2-well resonant-phonon design for a clean quantum level structure, in which the depopulation energy is significantly higher than one longitudinal phonon. In this study, various depopulation energies (small and large) are engineered in a 2-well design; the effect of the high-lying nonrelevant levels on the currents are systematically studied by using the non-equilibrium Green’s function method. The engineering of the depopulation energy is unable to avoid the formation of leakage channels, which are activated within at least three neighboring periods via sequential close tunneling. However, a large depopulation energy relaxes the thermal backfilling process; as a result, the net leakages at high temperatures can be significantly suppressed. In addition, pre-alignment remains a critical issue in the design when using a large depopulation energy, which requires improved engineering for the barriers to obtain better laser dynamics.

## Introduction

The terahertz (THz) electromagnetic spectrum (λ: 600–30 μm, *ħω*: 2–40 meV) remains the most underdeveloped, although several scientific and commercial applications have been demonstrated within these spectral ranges^1–3^. The most challenging issue is the lack of coherent and compact THz radiation sources with high output powers. THz quantum cascade lasers (THz-QCLs) are arguably treated as the only solid-state THz source that can deliver average optical power levels significantly greater than the milliwatt level^4^. These laser sources are not limited by the bandgap of semiconductor materials, and the working principle relies on the quantum tunneling in the hundreds of identical stacked periods via intersubband transitions (ISBT). Therefore, the THz frequency can be freely tuned, solely by engineering the energy separations of dispersed quantum levels. Rapid developments in THz-QCLs have been reported since they were first discovered in 2002^5^, which have covered lasing frequency ranging from 1.2 to 5.5 THz^4,6,7^ (when operated without the assistance of an external magnetic field). However, the maximum operating temperature (T_*max*_) remains below room temperature (< 300 K), thus requiring additional cooling components in real applications. An optimal T_*max*_ of approximately 200 K was reported in 2012^8^, which was not further renewed until 2019, when it was improved once again to 210 K^9^. A breakthrough of 250 K for T_*max*_ was reported in 2020^10^, indicating the possibility of achieving room-temperature operation (T_*max*_ of 300 K). All the high T_*max*_ values were achieved under a pulse operation mode and were based on metal–metal waveguides.

The THz radiation is mostly designed below the longitudinal (LO) phonon energy (approximately 35 meV) in GaAs materials. LO-phonon scattering has been treated as the main limiting degradation process at high temperatures, which contributes to the thermally activated parasitic channels among the relevant levels (non-radiative phonon resonance at the active regions instead of radiative photon emission)^11–14^, thermal backfilling^15,16^, or leakages into the high-lying non-relevant levels or continuums (hot-phonon assisting up-scattering from the upper-laser level)^17–19^. Meanwhile, broadening of the thermal radiation linewidth in an optical gain is also significant owing to the LO-phonon scattering along with other elastic- and inelastic-scattering, especially at high doping levels^20,21^. The corresponding strategies have been proposed to counteract the aforementioned thermal degradations, where the design parameters are actual trade-offs of one another. By recalling the historic increase of T_*max*_, the periodic quantum structures were actual continuously simplified^22–24^ until a 2-well configuration with a minimum of three levels was obtained from which one level was selected for resonant tunneling injection^9,10^.

Considering the aforementioned thermal degradations in the 2-well design, (a) a strong diagonality of ISBT between the two laser levels is engineered with an oscillator strength of approximately 0.3 (0.31^9^ and 0.33^10^), which is validated to suppress the non-radiation of LO-phonon emission. The diagonal radiation transition has been accepted as a common strategy for achieving the high T_*max*_. (b) The injector barrier needs to be carefully controlled to ensure an efficient resonant tunneling process, while enhancing the injection selectivity by reducing the parasitic coupling between the injection level and the lower-laser level. In addition, there are two critical features in the 2-well design; first, the barriers is significantly taller than those previously utilized^25,26^, such as Al_0.25_Ga_0.75_As^9^ and Al_0.3_Ga_0.7_As^10^ instead of Al_0.15_Ga_0.85_As. This deeply confines the electrons on the relevant levels, thus suppressing the carriers leaking directly over the barriers into the continuum area at high temperatures. The second key feature is the depopulation via intra-well LO-phonon resonance with relatively high energy; for example, 42 meV^9^ and 52 meV^10^. In comparison, the previous designs normally employ a depopulation energy that exactly equals to one LO phonon (~ 35 meV). Noted here, the aforementioned parameter values (radiation diagonality, anticrossing energy, and the depopulation energy) are from the recalculation of the structure obtained from references^9,10^ based on our models.

In this study, we introduce the nonequilibrium Green’s function (NEGF) method, which demonstrates the advances in the coherent effects and quantum mechanical dephasing, for analyzing the high-lying nonrelevant levels in a 2-well design. The depopulation energy is particularly tuned to be low or high. This study emphasizes any parasitic interactions within neighboring periods owing to an inherent narrow periodic length and a strong operating electric-field in this 2-well design. It demonstrates that the channels that formed from the relevant levels into the next two downstream periods can significantly affect the operating currents and pre-alignment current, which is more critical for designs with a large depopulation energy.

## Results and discussion

**Figure 1:**
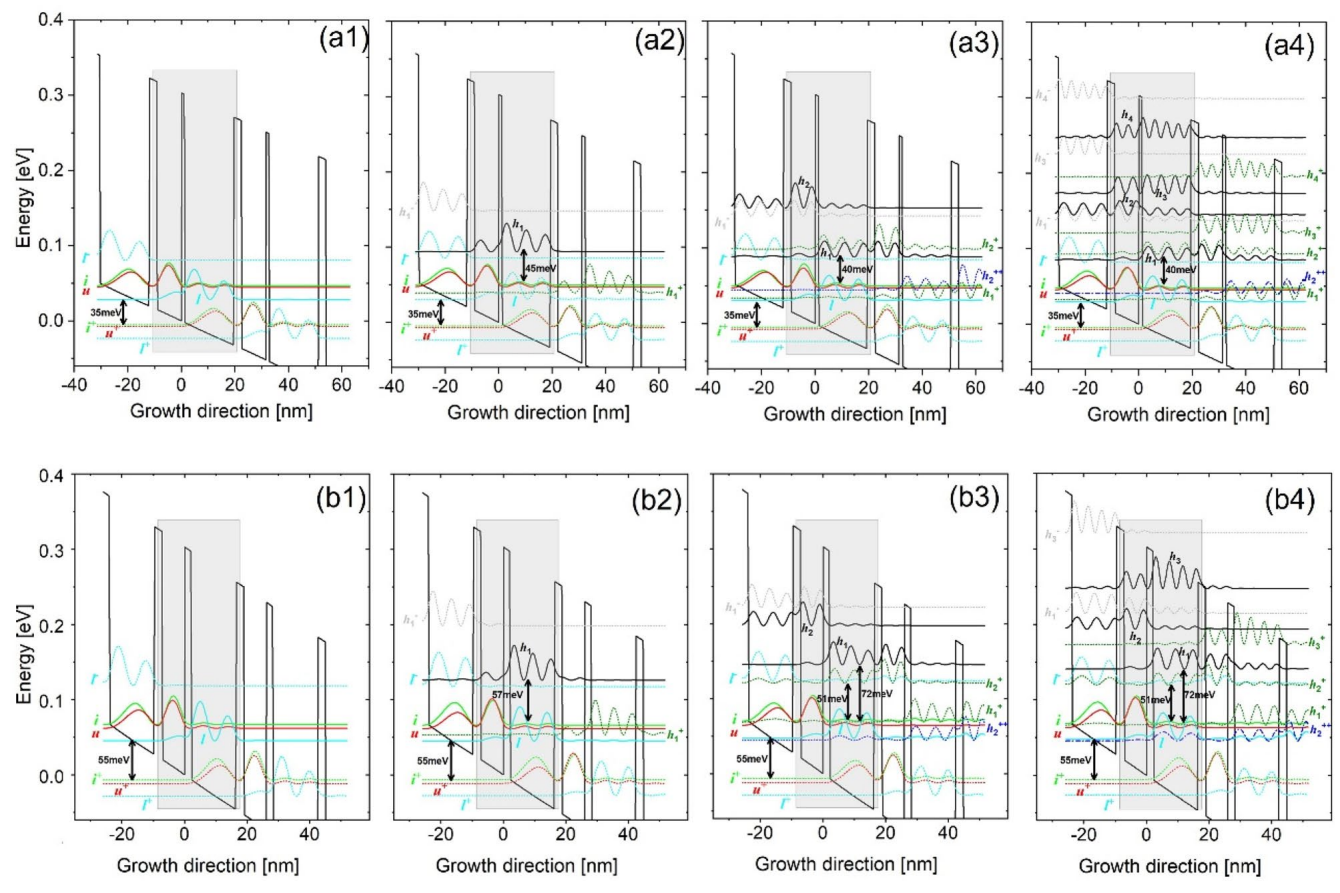
Conduction band profile of the investigated THz-QCLs employing the 2-well resonant-phonon scheme under an operating bias. The Wannier–Stark levels are indicated in three neighboring periods. Two depopulation energies are engineered; a 35 meV design (Al_0.3_Ga_0.7_As/GaAs, barriers/wells: **1.16**/18.4/**2.85**/9.16 nm) (**a1**–**a4**) and 55 meV design (Al_0.3_Ga_0.7_As/GaAs, barriers/wells: **2.1**/14.3/**2.5**/7.19 nm) (**b1**–**b4**). The underlined layer is doped by silicon at a sheet doping level of 3.2 × 10^10^ and 5.4 × 10^10^ cm^−2^, respectively. The conduction band offset is assumed as 300 meV. The THz photon emission energy is 16 meV. The axial cut-off energies are tuned by controlling the exact number of quantum levels in the calculations. (**a1**,**b1**) contains only three relevant levels, (*i*,*u*,*l*)-scale; (**a2**,**b2**) contains one more nonrelevant level along with the relevant levels, (*h*_1_)-scale; (**a3**,**b3**) contains two more nonrelevant levels along with the relevant levels, (*h*_1_,*h*_2_)-scale; (**a4**,**b4**) contains all the nonrelevant levels until the continuum area, (*full*)-scale. Noted that the different energy scale can slightly effect on the relevant levels (i.e., injector alignment etc.), therefore the realignment at injector is performed for each scale.

**Figure 2:**
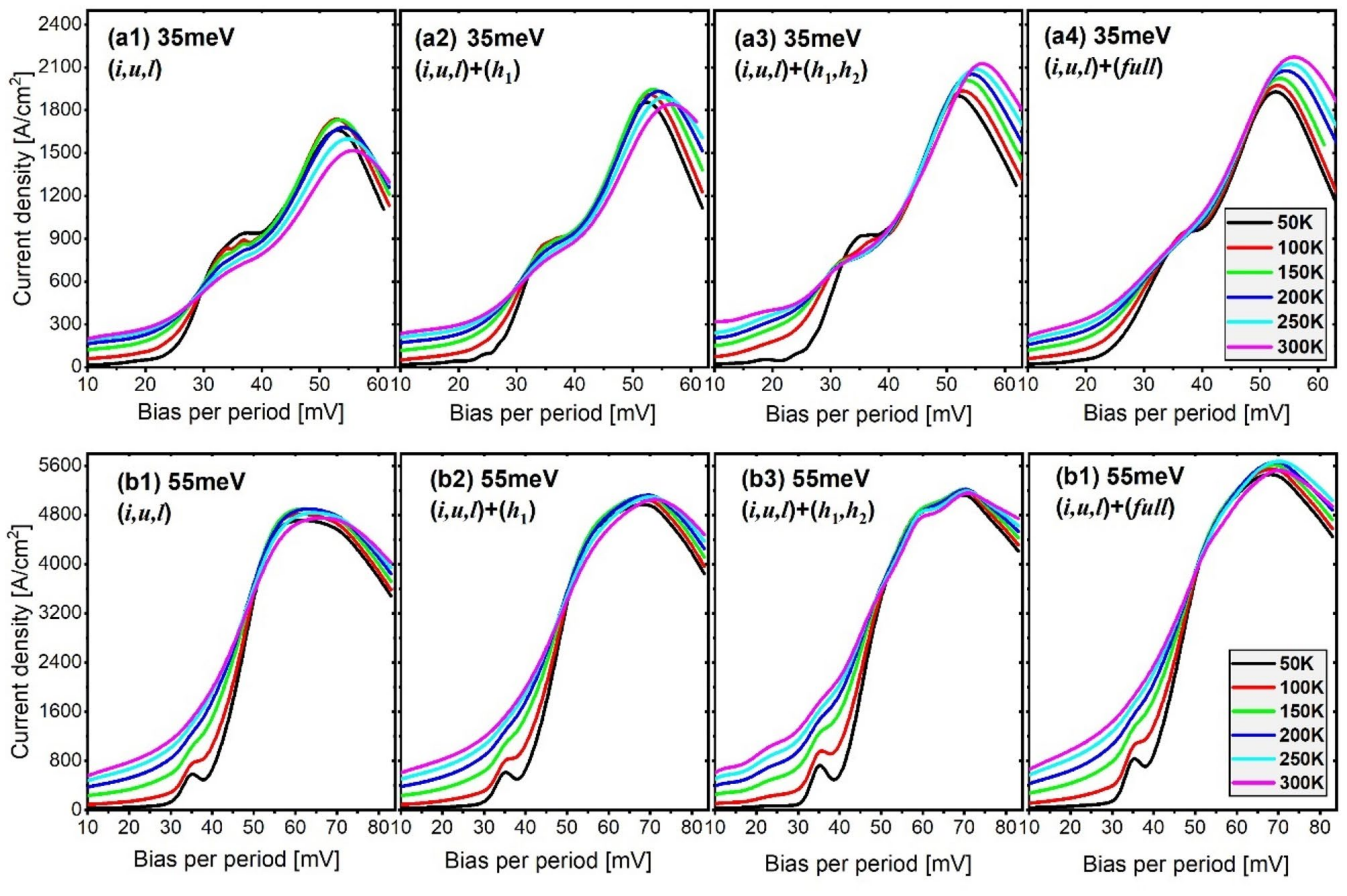
Current–voltage curves at different temperatures as a function of the applied bias for both the 35 meV design (**a1**–**a4**) and 55 meV design (**b1**–**b4**). Each fig represents the curves at different level scale.

**Figure 3:**
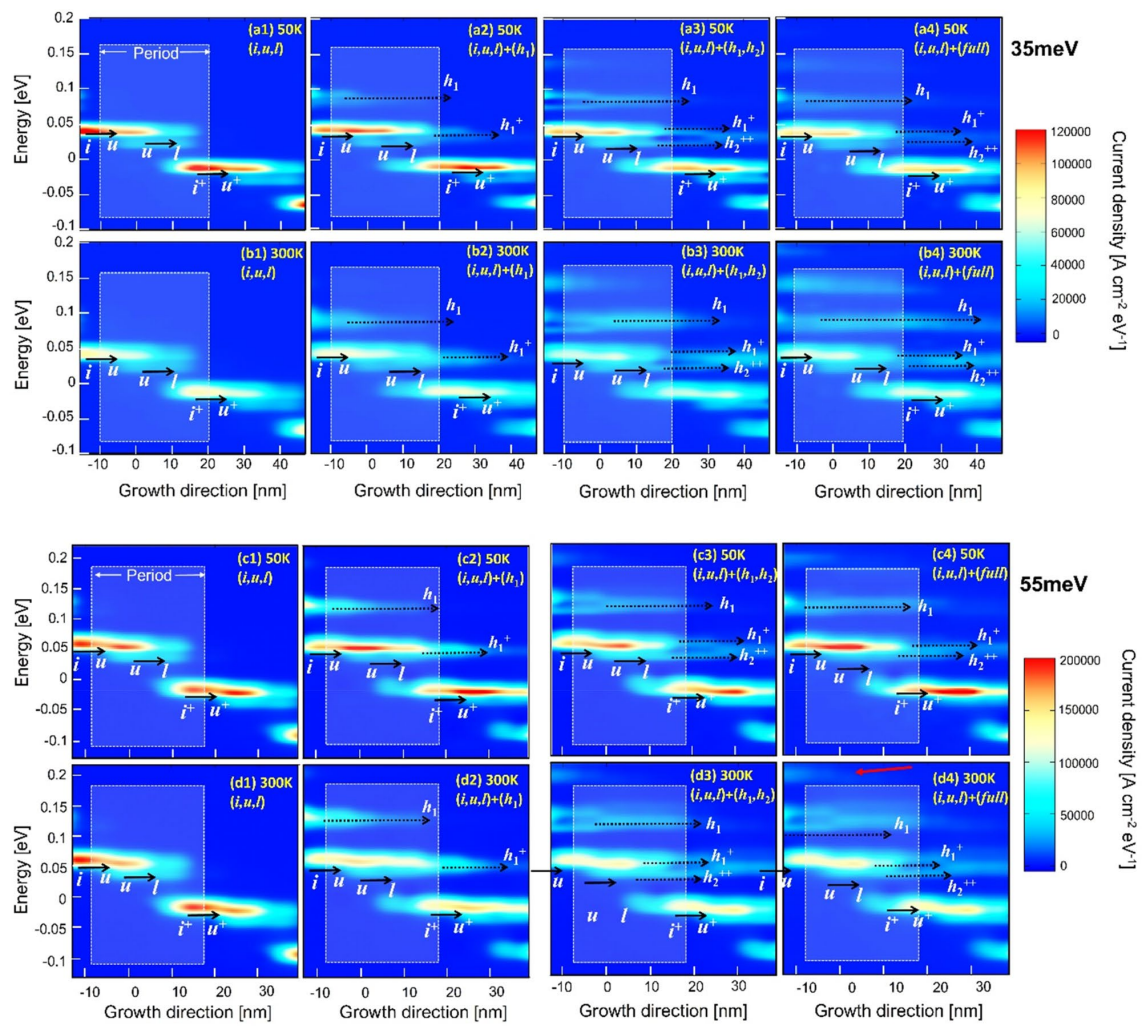
The spatial and energy resolved current mappings under the operating bias condition at low and high temperatures (50 K and 300 K). Figures (**a1**–**a4**) and (**b1**–**b4**) indicate the mappings for the 35 meV design; Figures (**c1**–**c4**) and (**d1**–**d4**) indicate the mappings for the 55 meV design.

By simply tuning the axial cut-off energy range, the exact number of quantum levels involved in a calculation can be controlled. As shown in Fig. 1, only three basic quantum levels are included (injector level *i*, upper laser level *u*, and lower laser level *l*), that is, the (*i*,*u*,*l*)-scale. The other scales include more high-lying nonrelevant levels, such as the (*h*_1_)-scale, (*h*_1_,*h*_2_)-scale, and finally extends to the continuums as a (*full*)-scale. The tall barriers of Al_0.3_Ga_0.7_As are selected in this study; the working principle of this 2-well design is presented herein. First, the electrons traverse through the cascading levels along the growth direction by the tunneling mechanisms. The tunneling probability is enhanced at the injector for a bias at which levels *i* and *u* across a potential barrier are at the same energy, which then makes the tunneling a resonant process for achieving an efficient injection. At the active region, the population is inverted between levels *u* and *l*, where a diagonal ISBT radiation is accomplished. At the extractor region, the depopulation of level *l* follows an intra-well LO-phonon resonance (direct-phonon) by designing the energy separation between level *l* and the following injector *i*^+^ to be equal to 35 meV (Fig. 1a, 35 meV design) or significantly larger than one LO phonon at 55 meV (Fig. 1b, 55 meV design). This depopulation energy can be achieved by simply tuning the thickness of the lower-well, where the total period length of the 35 meV design is 31 nm, and is 16% narrower at 26 nm for the 55 meV design. Three neighboring cascading periods are shown in the Fig. 1 [upper minus (−) for the upstream period levels; upper plus (+) for the downstream period levels]. The currents flowing across each period as a function of the applied electric field (I–V curves) at different temperatures in both designs are presented in Fig. 2. Based on these I–V curves, the maximum current J_*max*_, the current leaking through the nonrelevant levels J_*leak*_, and the pre-alignment current J_*p*_ are extracted and studied. Figure 3 demonstrates the corresponding current mappings that were resolved by space and energy under the different scales at low and high temperatures (50 K and 300 K).

### Tendency of maximum current J_***max***_

The J_*max*_ conditions are normally obtained at an operating bias where the greatest contributions are from the resonant tunneling at the injector. A large coupling energy *ħ*Ω at the injector truly profits the injection current, and leads to an efficient injection for level *u*; however, when the resonance splitting is strong enough, the parasitic channel between levels *i* and *l* will be significantly enhanced, as a result, the injecting selectivity is destroyed. In addition, an excessive splitting also can deform the laser gain profile shape and decay the gain magnitude. For a THz-QCL, the splitting of the resonant tunneling at the injector is between 2 and 3 meV; this balanced value is apparently smaller than the quantum level broadenings (10–20 meV). As a result, the resonant tunneling process undergoes strong dephasing. Here, we first studied the tendency of J_*max*_ by considering only the three basic levels, and then used this information as a fundamental standard for further analysis after including more high-lying nonrelevant levels. In the (*i*,*u*,*l*)-scale, J_*max*_ is expressed by the resonant tunneling current at the injector as follows^27^:

1 $${J}_{max} \sim en*\left(\frac{2{\Omega }_{iu}^{2}{\tau }_{\parallel }}{4{\Omega }_{iu}^{2}{\tau }_{u}{\tau }_{\parallel }\left[1+\frac{{\tau }_{l{i}^{+}}}{2{\tau }_{ul}}\right]+1}\right)$$

where *n* is the doping density in each period, 2*ħ*Ω is the energy splitting at the anticrossing between levels *i* and *u* at an alignment condition, which is exponential to the thickness of the injector barrier. τ_*u*_ is the lifetime of level *u*, that is, 1/τ_*u*_ = 1/τ_*ui*_ + 1/τ_*ul*_. τ_*ul*_ indicates the scattering time from level *u* to *l*, and τ_*li*+_ is the scattering time from level *l* to the next injector *i*^+^. τ_‖_ is the dephasing time in this resonant tunneling process^27^. The ratio τ_*li*+_/2τ_*ul*_ in Eq. (1) can be ignored at low temperatures; meanwhile, the dephasing time τ_‖_ is significantly high, therefore the J_*max*_ can be simply expressed by J_*max*_ ~ 1/τ_*u*_. Following this simplified expression, J_*max*_ will show an increasing tread as the temperature increases, that is because the τ_*u*_ becomes smaller thanks to the thermally enhanced non-radiation channels at the level *u*. However, when the temperature reaches up to a certain level, the dephasing time τ_‖_ will be sufficiently reduced and become a determinant factor for J_*max*_, where Eq. (1) will then be expressed by J_*max*_ ~ τ_‖_. As a result, when the temperature raises up further, J_*max*_ starts to decrease and presents a turnover. Noted here, the ratio τ_*li*+_/2τ_*ul*_ in Eq. (1) also becomes large at high temperatures; for the 35 meV design, τ_*li*+_/2τ_*ul*_ is 12 at 50 K and 6 at 300 K, and for the 55 meV design, τ_*li*+_/2τ_*ul*_ is 16 at 50 K and 9 at 300 K. The net change in the τ_*li*+_/2τ_*ul*_ ratio is relatively small (in one order), it will only slightly shift the appearance of the turnover point and does not eliminate the turnover trend.

**Figure 4:**
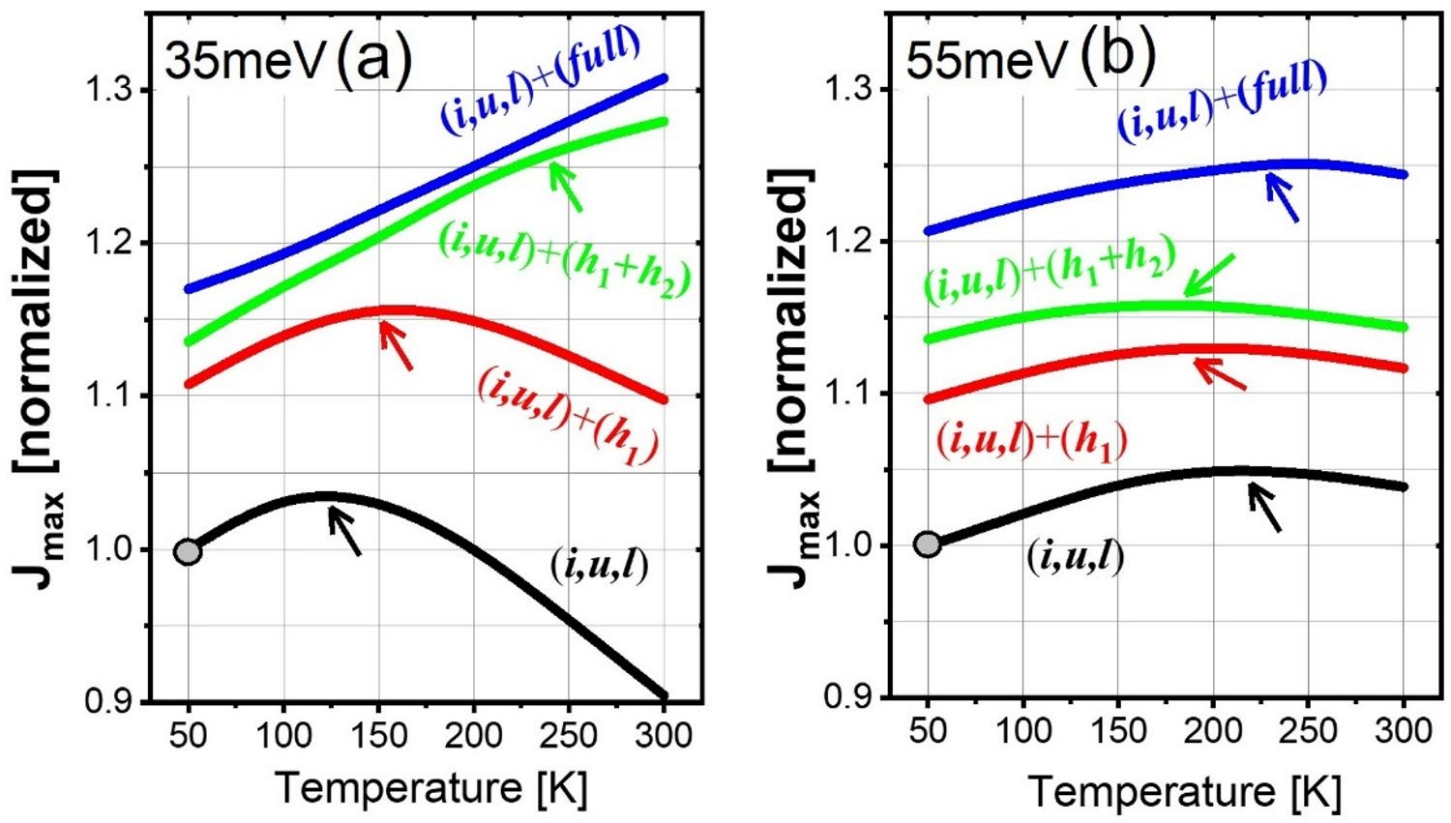
J_*max*_ as functions of temperatures under different scale levels for both the 35 meV design (**a**) and 55 meV design (**b**). J_*max*_ in the (*i*,*u*,*l*)-scale at 50 K is normalized as 1 and used as a standard.

The (*i*,*u*,*l*)-scale J_*max*_ in both the 35 meV and 55 meV designs is shown in Fig. 4a,b by the solid black curves, where the y-axis is normalized by the J_*max*_ at 50 K (marked as the gray circle). It is clear that the (*i*,*u*,*l*)-scale J_*max*_ plots demonstrate turnovers for both designs; however, the appearance of the turnover occurs much earlier in the 35 meV design than that in the 55 meV design (125 K < 210 K, indicated by black arrows). Meanwhile, beyond the turnover point, the decline of (*i*,*u*,*l*)-scale J_*max*_ is significantly sharper in the 35 meV design, whereas a slow decrease is maintained in the 55 meV design. This different J_*max*_ trends regarding the turnover point and the afterward decrease can be explained by the varying coupling status at the injector for both designs; that is, for resonant tunneling, the 55 meV design shows the splitting energy of 4.5 meV at the injector, which is nearly 2 times that of in the 35 meV design. Now, we move to see how the J_*max*_ trends depending on the temperature change after including the high-lying nonrelevant levels. It is obvious the trend of J_*max*_ became significantly different from the (*i*,*u*,*l*)-scale. Considering the turnover points (Fig. 4a,b, color arrows), the inclusion of only one nonrelevant level *h*_1_ (red curves) hardly changed the turnover points in both designs, and only leads to a net 10% increasing of the J_*max*_; this is because the resonant tunneling process at the injector between levels *u* and *l* is nearly undisturbed by this single nonrelevant level *h*_1_. When more nonrelevant levels are considered, such as the (*h*_1_,*h*_2_)-scale or (*full*)-scale, the turnovers of J_*max*_ disappears in 35 meV_design, and the J_*max*_ continuously increases in the whole temperature range from 50 to 300 K. In comparison, for the 55 meV design, the turnover in J_*max*_ remains regardless of whether the nonrelevant levels are included or excluded.

The departure of the J_*max*_ turnover trend due to more nonrelevant levels in two designs can be explained by Fig. 1 (a) in the 35 meV design, in case of (*h*_1_,*h*_2_)-scale (Fig. 1a3), *h*_1_ forms a sequential close tunneling channel with the upstream period relevant level *l* (*h*_1_^+^ ↔ *l*) (slightly misaligned), and *h*_2_ in the downstream period can enhance this channel by extending it towards the growth direction. Level *h*_2_ simultaneously opens an additional sequential tunneling channel with the pre-upstream period relevant level *u* (two periods up) (*h*_2_^++^ ↔ *u*). Considering the coupling for these two channels (*h*_1_^+^ ↔ *l*; *h*_2_^++^ ↔ *u*), Fig. 1a2,a3 demonstrates that if only level *h*_1_ is included, because it is exactly centered between the upstream period levels *u* and *l*, *h*_1_ couples with them weakly (*h*_1_^+^ ↔ *u/l*). However, when both levels *h*_1_ and *h*_2_ are simultaneously included, first, level *h*_1_ couples strongly with the downstream period level *h*_2_ thanks to the near alignment in energy between them (*h*_1_^+^ ↔ *h*_2_^++^), that the splitting energy between these levels is approximately 7 meV. This large splitting causes level *h*_2_ approaching the pre-upstream period level *u* (*h*_2_^++^ ↔ *u*), and level *h*_1_ approaching level *l* in the same pre-upstream period (*h*_1_^+^ ↔ *l*). Figure 1a3 demonstrates that the levels *h*_2_^++^ and *u* are spatially distant as they are separated by nearly two period lengths, > 50 nm. As a result, the coupling of *h*_2_^++^ ↔ *u* will be weak. Comparatively, for the other channel *h*_1_^+^ ↔ *l*, level *h*_1_^+^ is spatially close to level *l* (a separation of only 11 nm). Meanwhile, the detuning energy between *h*_1_^+^ and *l* is only 0.8 meV; as a result, the splitting energy between these levels is large as 1.5 meV. Thus, channel *h*_1_^+^ ↔ *l* can be concluded to be more efficient than *h*_2_^++^ ↔ *u*. In particular, leakages at high temperatures across channel *h*_1_^+^ ↔ *l* will be more significant because the thermal backfilling from the injector area into level *l* (*i*^+^ → *l*) can be more serious at high temperatures; Based on a quantification, the thermal backfilled population at level *l* is as high as 16% share of the total population at 300 K. (b) The levels distribution in 55 meV design is similar only if level *h*_1_ is considered, where *h*_1_ also positions at the center of the upstream period levels *u* and *l* (*h*_1_^+^ ↔ *u/l*), as shown in Fig. 1b2. After both levels *h*_1_ and *h*_2_ are included (Fig. 1b3), levels *h*_1_^+^ and *h*_2_^++^ can form a significantly strong coupling, i.e., the detuning energy between them is 0.5 meV, and the corresponding splitting energy is 13 meV, almost two times of that in 35meV_design. This strong coupling forces level *h*_1_^+^ to approach level *u*, and *h*_2_^++^ to approach level *l*, thus finally forming the double channels similar to the 35 meV design. Noted here, both channels are indeed under strong resonance tunneling conditions (Fig. 1b3), in which the current leakages via both channels can be determined by dephasing. This can explain the (*h*_1_,*h*_2_)-scale and (*full*)-scale J_*max*_ remaining turnover phenomenon in the 55 meV design, as shown in Fig. 4b.

In addition, even at room temperature of 300 K, in the 55 meV design, the increasing of a (*full*)-scale J_*max*_ is only 1.2 times as compared with the (*i*,*u*,*l*)-scale J_*max*_, where a increasing is even 1.5 times in the 35 meV design. It is ascribed to the suppression of the thermal backfilling owing to the large depopulation energy. For the 55 meV design, the thermal backfilled population from the injector area (*i*^+^) into level *l* is a 9% share of the total population at 300 K, which is much lower than a 16% share in the 35 meV design. The current mappings in Fig. 3a3,b3 and c3,d3 demonstrate the current leaking through the channels. In addition, differing from the almost overlapping of the (*h*_1_,*h*_2_)-scale and (*full*)-scale J_*max*_ curves in the 35 meV design (green and blue curves in Fig. 4a), for the 55 meV design, the (*full*)-scale J_*max*_ curve is apparently larger than that of the (*h*_1_,*h*_2_)-scale. The reason is, despite the Al_0.3_Ga_0.7_As tall barriers are used, it still cannot avoid the leakages directly into the continuum area in the 55 meV design. The sequential tunneling leakage can be extended out of the confined areas into the continuum, as shown in the current mapping presented in Fig. 3d4 (indicated by red arrow). This gives evidence that, for the 2-well design with a large depopulation energy, the Al_0.3_Ga_0.7_As barriers are not tall enough and further taller barrier, such as Al_0.35_Ga_0.65_As, may be required.

### Tendency of leakage current J_***leak***_

**Figure 5:**
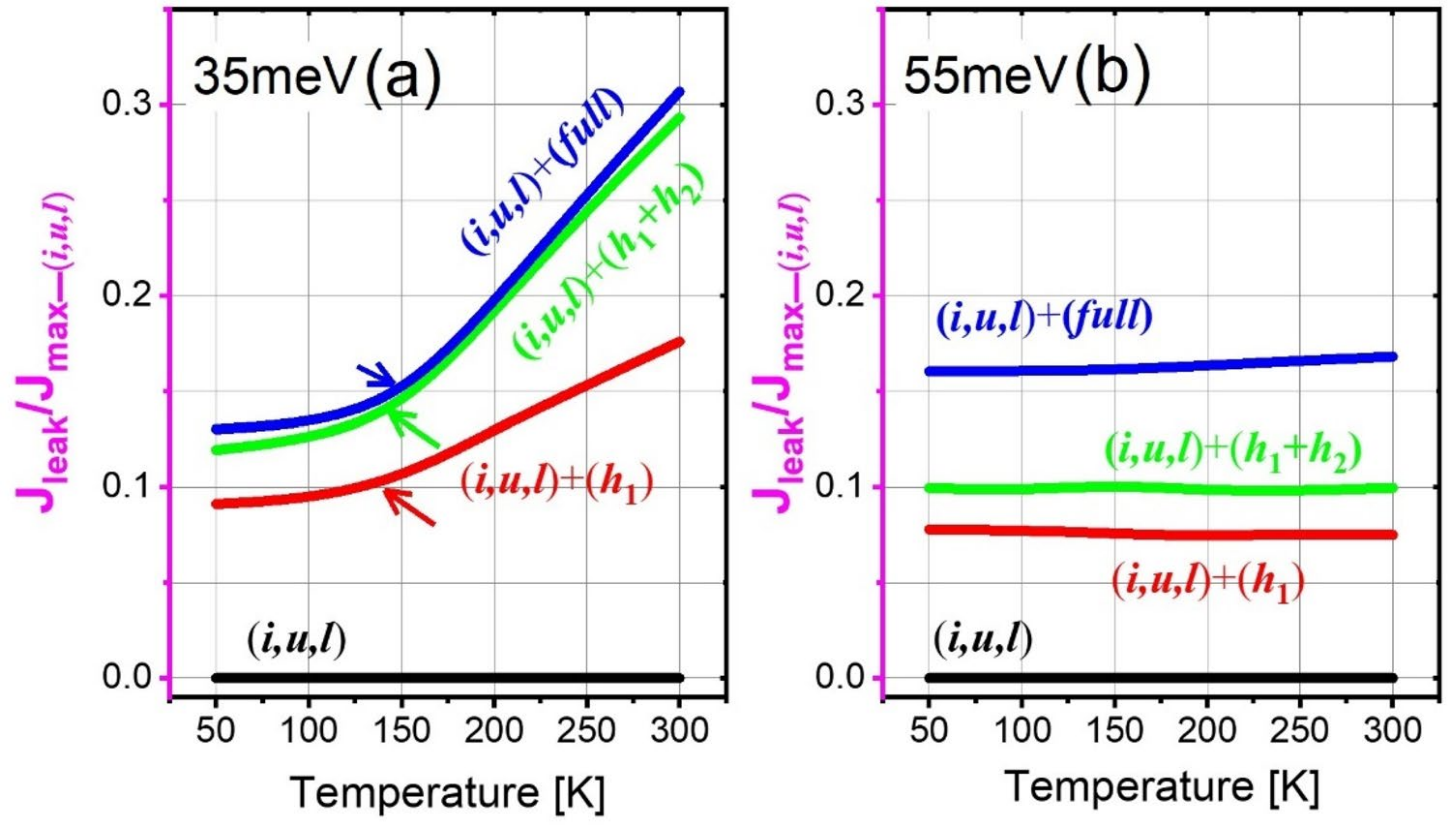
The J_*leak*_/J_*max*_ ratio as functions of the temperatures under different scale levels for both the 35 meV design (**a**) and 55 meV design (**b**). J_*max*_ in the (*i*,*u*,*l*)-scale is normalized as 1 and used as a standard. There are no leakages across the nonrelevant (*i*,*u*,*l*)-scale, this ratio is 0, as indicated by the black solid curves.

Figure 5a,b demonstrates the magnitude of J_*leak*_ across the nonrelevant levels which is quantified by the ratio of J_*leak*_/J_*max*_. We define J_*leak*_ as the difference between the current calculated and the current restricting to the three relevant levels. Here, the (*i*,*u*,*l*)-scale J_*max*_ (without any effect from nonrelevant levels) is set as the normalized standard at 1; As there are no leakage currents through the nonrelevant levels in this (*i*,*u*,*l*)-scale, J_*leak*_/J_*max*_ is 0, as indicated by the black lines in Fig. 5. (a) For the 35 meV design, at a low temperature of 50 K, the J_*leak*_/J_*max*_ ratio is ~ 0.1 when only level *h*_1_ is included. When more levels are considered, i.e., (*h*_1_,*h*_2_)-scale and (*full*)-scale, the J_*leak*_/J_*max*_ ratio is ~ 0.12. At a high temperature of 300 K, the J_*leak*_/J_*max*_ ratio becomes much higher; that is, the J_*leak*_/J_max_ ratio is ~ 0.17 when only considering level *h*_1_, and increases to ~ 0.3 when more levels are considered. The (*h*_1_,*h*_2_)-scale and (*full*)-scale J_*leak*_/J_*max*_ ratio tends to nearly overlap, which denotes that the main nonrelevant levels effecting the leakages are *h*_1_ and *h*_2_. In addition, based on the slope of J_*leak*_/J_*max*_ demonstrated in Fig. 5a, a sharp increase in this ratio occurs at temperatures > 150 K, this is consistent with the thermal backfilling effect that is significant at temperatures above 150 K. The enhanced leakages towards the growth direction are apparent at high temperatures, as shown in the current mapping in Fig. 3b3,b4. (b) For the 55 meV design, as shown in Fig. 5b, the J_*leak*_/J_*max*_ ratio remains approximately constant and independent of the temperatures. The constant trend of this ratio once again indicates the suppression of thermal backfilling by using a larger depopulation energy in a large depopulation energy 55 meV design.

### Tendency of the pre-alignment current J_***p***_

**Figure 6:**
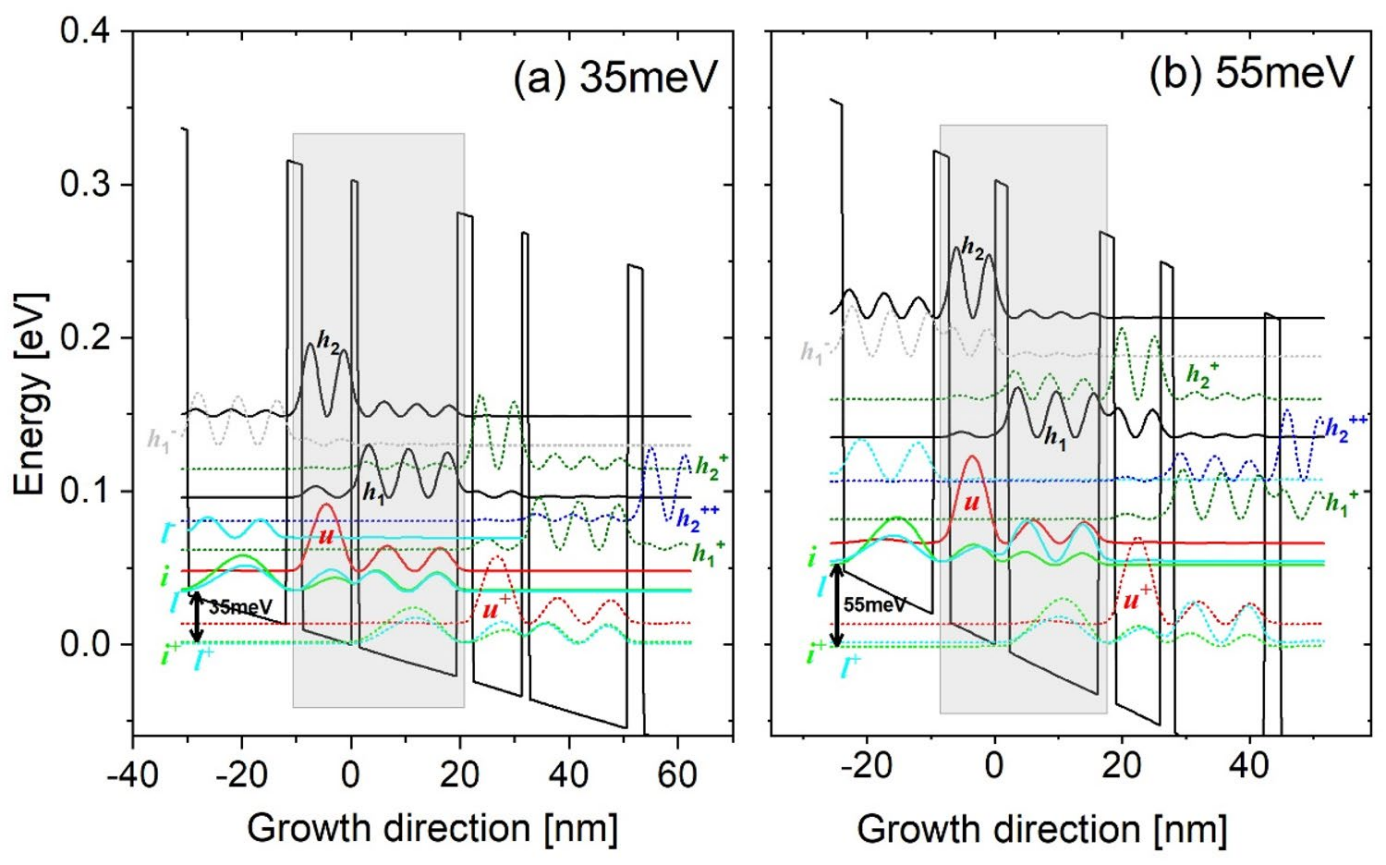
The pre-alignment condition where the injector level *i* aligns with level *l*, (**a**) 35 meV design; (**b**) 55 meV design.

**Figure 7:**
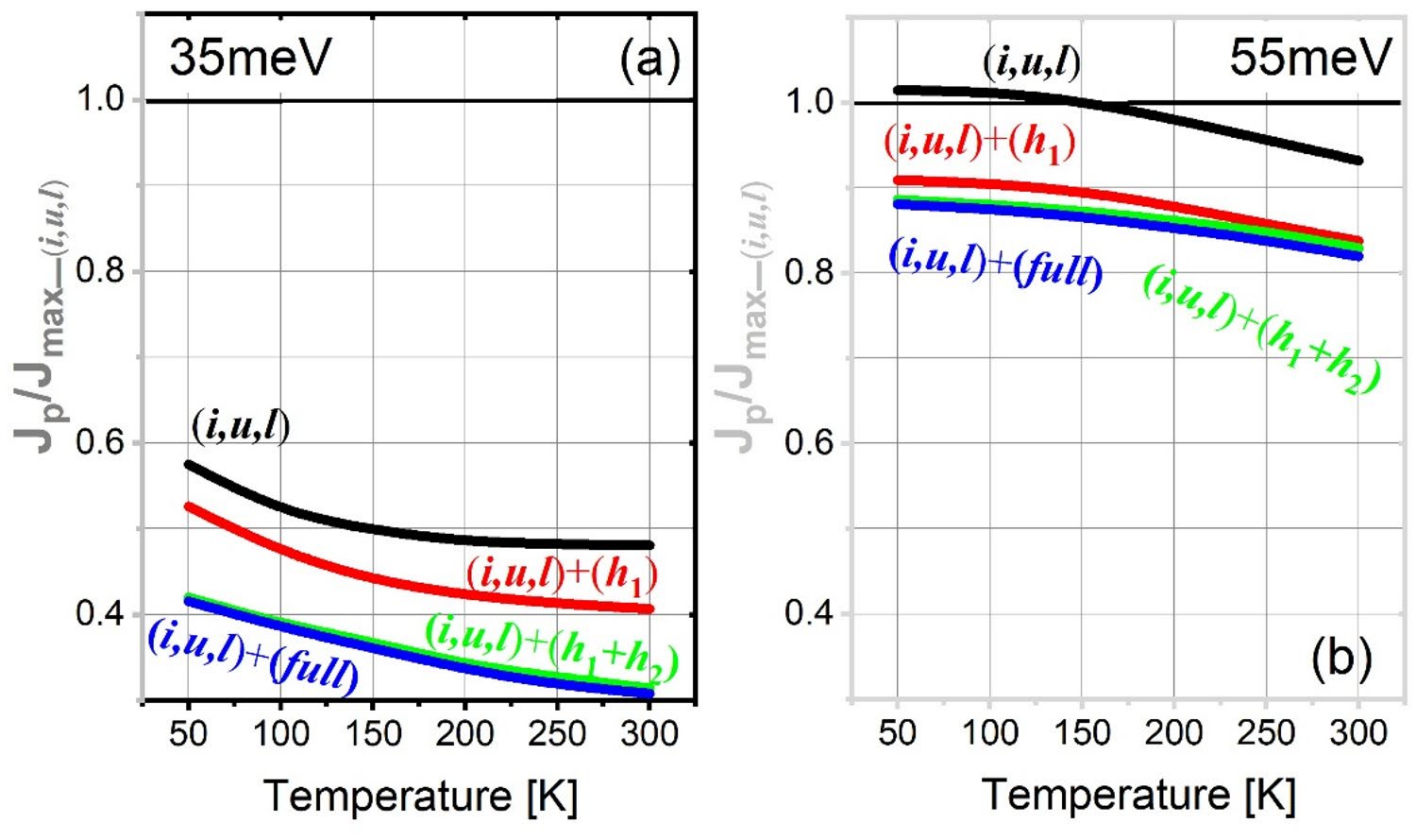
The J_*p*_/J_*max*_ ratio as functions of the temperatures under different scale levels for both the 35 meV design (**a**) and 55 meV design (**b**).

**Figure 8:**
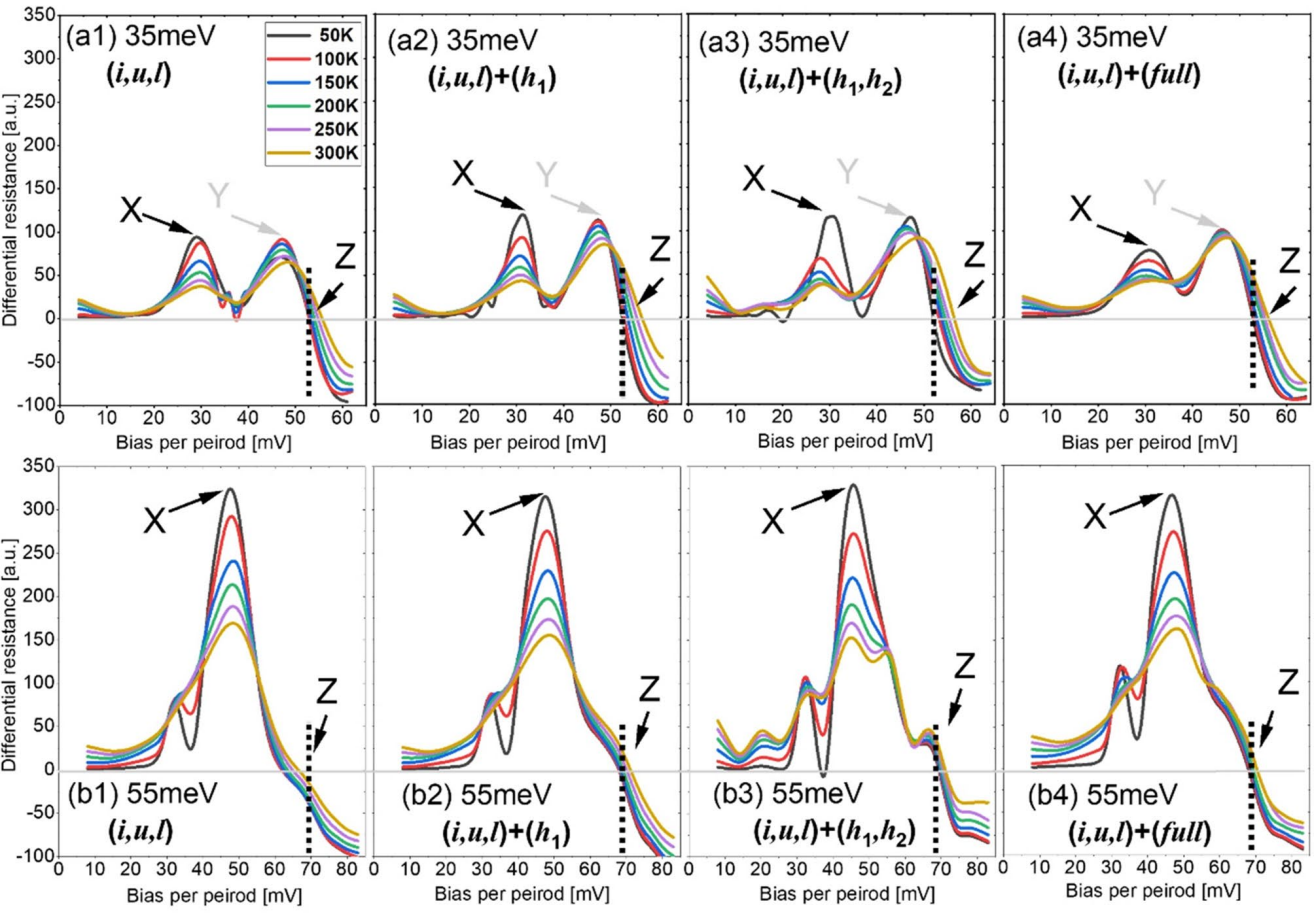
The differential resistances as functions of the bias at different temperatures, 35 meV design (**a1**–**a4**), 55 meV design (**b1**–**b4**). The X-peak indicates the pre-alignment condition, the Y-peak in figs (**a1**–**a4**) is the peak in the bias regime between the pre-alignment and operation, Z indicates the operating bias condition.

Pre-alignment of the injector level *i* and the lower-laser level *l* at the bias before the operating bias usually forms a strong parasitic channel, thus possibly creating the negative difference resistance (NDR) in the I-V characteristics in the bias regime with a positive gain^28^. The electrical instabilities in THz-QCLs may occur owing to this NDR. A positive population inversion (leading to optical gain) is usually established by passing this pre-alignment condition. Therefore, it is critical to study the current at the pre-alignment condition J_*p*_ when different designs are proposed. Figure 6 presents this pre-alignment condition in both the 35 meV and 55 meV designs in this study. Here, we use the J_*p*_/J_*max*_ ratio to represent the effecting magnitude of this pre-alignment, as shown in Fig. 7. At the same time, we also study the differential resistance depending on the applied bias (as shown in Fig. 8). First, this pre-alignment current J_*p*_ is expressed at the bias where level *i* and level *l* algin^27^:

2 $${J}_{p} \sim en*\frac{2{\Omega }_{il}^{2}{\tau }_{\parallel }}{1 + 4{\Omega }_{il}^{2}{\tau }_{\parallel }{\tau }_{l}}$$

By recalling the Eq. (1), it is stated that the lifetime of the tunneled-end level (upper-laser level *u* in Eq. (1), τ_*u*_) dominates the current at low temperatures, and the dephasing time dominates the current at high temperatures, a turnover of the resonant tunneling current clearly exists. This is mainly due to the assumptions of $4{\Omega }_{il}^{2}{\tau }_{\parallel }{\tau }_{u}$ >> 1 at low temperatures and << 1 at high temperatures. However, in Eq. (2), these assumptions cannot be made for $4{\Omega }_{il}^{2}{\tau }_{\parallel }{\tau }_{l}$ because the τ_‖_ and τ_*l*_ are always opposite regardless of the temperatures. At low temperatures, the dephasing time τ_‖_ is large; however, τ_*l*_ is significantly small thanks to ultrafast LO-phono resonance at level *l*. At high temperatures, τ_‖_ becomes significantly small owing to the thermally enhanced scatterings; however, τ_*l*_ becomes large as a result of the thermal backfilling. However, we can assume that $4{\Omega }_{il}^{2}{\tau }_{\parallel }{\tau }_{u}$ has a small variation range. Therefore, the J_*p*_ is considered to be solely dominated by the dephasing time τ_*‖*_.

- *J*_*p*_*/J*_*max*_*:* in the 35 meV design, the J_*p*_/J_*max*_ ratio is small below 0.6 and decreases as temperature increases in all scale cases (Fig. 7a); in details, for the (*i*,*u*,*l*)-scale, the ratio is 0.58 at 50 K and 0.48 at 300 K. Similar trends are observed after including the nonrelevant levels, however, the net ratio will be smaller; for examples, in the (*h*_1_,*h*_2_)-scale and (*full*)-scale, the ratio is 0.41 at 50 K and 0.3 at 300 K. In comparison, in the 55 meV design, the J_*p*_/J_*max*_ ratio is much higher (Fig. 7b). First, in the (*i*,*u*,*l*)-scale, the ratio is even greater than 1 at low temperatures < 150 K. When the nonrelevant levels are included, this ratio is lower than 1 but remains greater than 0.8 regardless of the temperatures. Therefore, it can be summarized here: First, for both the 35 meV and 55 meV designs, this pre-alignment current J_*p*_ is more serious at low temperatures (i.e., a larger ratio). Second, the inclusion of nonrelevant levels can reduce this J_*p*_/J_*max*_ ratio, which may contribute beneficially to the laser dynamics. Third, pre-alignment may be critical for designs with a larger depopulation energy.
- *Differential resistance:* Fig. 8 demonstrates the differential resistance depending on the applied bias at different temperatures. First, besides the 55 meV design with the (*i*,*u*,*l*)-scale, for all scale levels, there are no NDRs before the operating bias condition (Z, as shown in Fig. 8) (*y*-axis differential resistance < 0). An apparent NDR only occurs at a bias region beyond the operation, which has been commonly observed in the 2-well designs^9,10^. The X-peak of the resistance in Fig. 8 indicates a pre-alignment, the resistance at this X-peak decreases apparently as the temperature increases from 50 to 300 K in both designs. For the 35 meV design, an obvious second peak (Y) emerges before the operating bias condition (Z). This indicates that when the bias passes the pre-alignment, the current will increase rapidly first and slowly then. This Y-peak in resistance is not observed in the 55 meV design, and the resistance declines sharply after the X-peak. It needs to note that, the onset of lasing in a QCL can be observed by a slope discontinuity in its I–V characteristics^29^. Here, in the NEGF model, the stimulated radiation effecting on the current is not included; therefore, the discontinuity cannot be observed from the differential resistance’s plots in Fig. 8. In general, the lasing can slightly enhance the current.

### Quantum well structures for the 35 meV and 55 meV designs

A 2-well design with a large depopulation energy is achieved by narrowing the whole period length, indicating that the operating electric field becomes stronger; for example, the 55 meV design shows the electric field at 27.7 kV/cm, 1.56 times of that in the 35 meV design. The features of these stronger electric field and shorter period length will cause complicated inter-periodic parasitic coupling from the nonrelevant levels with the upstream relevant levels. In addition, high-T_*max*_ THz-QCLs usually have a total active region thickness of approximately 10 μm for a metal–metal waveguide structure, the total external bias applied will be much larger owing to the total number of stacked periods in a design with narrow periods, which may complicate the L–I–V measurement and device thermal management. As previously indicated, pre-alignment may be critical in the narrow-design with a large depopulation energy, which requires a more careful balance of the barrier thickness to engineer the inter-period parasitic couplings for improved laser dynamics.

## Conclusion

By introducing the self-consistent NEGF method, nonrelevant levels effecting the current in a 2-well resonant-phonon design are studied. Two designs with small and large depopulation energies (employing the different period lengths) are used. Nonrelevant levels can activate the sequential close tunneling channels at least in three neighboring periods. The dominant channel origins from the relevant level *l* and can be largely enhanced at high temperatures owing to the thermal backfilling from the electron pool at the injector. Although the coupling of this channel is stronger in a large depopulation energy design (55 meV design), it reduces the leakages because the thermal backfilling is sufficiently suppressed. When a tall barrier is used for both the 35 meV and 55 meV designs, the first and the second high-lying levels are the main effecting nonrelevant levels. In addition, a design with large depopulation energy requires more attentions for engineering the pre-alignment current, as the laser dynamic in such designs can be largely limited.

## Method

The calculation in this study is based on NEGF, which is also known as the Keldysh or Kadanoff-Baym formalism^30–32^, and we perform it in a cylindrical basis for the in-plane motion. It considers the quantum transports such as coherent/incoherent resonant tunneling, as well as multiple scattering mechanisms. Inelastic scatterings (optical and acoustic phonons) and elastic scatterings (charged impurities, interface roughness, and alloy disorder) are considered within the self-consistent Born approximation. In particular, the charged impurity scattering is treated within the full in-plane dependence thus reflecting the long-distance effect of the coupling to local impurities. For electron–electron interaction, it is assumed as the mean-field approximation by solving the corresponding Poisson equation self-consistently. In the NEGF formalism, the scattering processes are described in terms of the self-energy. Self-energies and Green’s functions are calculated in a self-consistent manner, as both elastic and inelastic scattering processes are accounted for within the self-consistent Born approximation^33–35^. The field-periodic boundary condition is set for the repeating cascading periods.

At the beginning of the calculations, the 3-band effective mass Schrödinger equation is solved in real space. The energy levels (minibands) are selected by controlling the axial energy cut-off. The minibands are then transformed into a localized basis of modes, which is called the reduced real space basis. These wave functions are then used as a basis in the NEGF algorithm. The models then enter the second step, and the scattering coupling terms are calculated for each of the accounted mechanisms^21,36,37^. The main part of the calculation is a self-consistent NEGF solver. It starts from an initial guess of the Green’s functions, and the self-energies are then approximately demonstrated. The Green’s functions are calculated iteratively. Simultaneously, the mean-field electrostatic potential is calculated self-consistently (Poisson’s equation). These iterations are performed until convergence is reached for the Green’s functions, as well as for the calculated current. Subsequently, the current density, as well as the carrier density distributions are determined.

## Data Availability

The datasets used and/or analyzed during the current study available from the corresponding author on reasonable request.
